# Tuberculosis in Antiretroviral Treatment Programs in Lower Income Countries: Availability and Use of Diagnostics and Screening

**DOI:** 10.1371/journal.pone.0077697

**Published:** 2013-10-17

**Authors:** Lukas Fenner, Marie Ballif, Claire Graber, Venerandah Nhandu, Jean Claude Dusingize, Claudia P. Cortes, Gabriela Carriquiry, Kathryn Anastos, Daniela Garone, Eefje Jong, Joachim Charles Gnokoro, Omar Sued, Samuel Ajayi, Lameck Diero, Kara Wools-Kaloustian, Sasisopin Kiertiburanakul, Barbara Castelnuovo, Charlotte Lewden, Nicolas Durier, Timothy R. Sterling, Matthias Egger, for the International epidemiological Databases to Evaluate AIDS (IeDEA)

**Affiliations:** 1 Institute of Social and Preventive Medicine, University of Bern, Bern, Switzerland; 2 Swiss Tropical and Public Health Institute, Basel, Switzerland; 3 University of Basel, Basel, Switzerland; 4 Centre for Infectious Disease Research in Zambia, Lusaka, Zambia; 5 Women's Equity in Access to Care & Treatment, Kigali, Rwanda; 6 University of Chile School of Medicine, Santiago, Chile; 7 Instituto de Medicina Tropical Alexander von Humboldt, Lima, Peru; 8 Montefiore Medical Center and Albert Einstein College of Medicine, Bronx, New York, United States of America; 9 Khayelitsha ART Programme, Médecins Sans Frontières, Cape Town, South Africa; 10 Faculty of Health Sciences, University of Witwatersrand, Johannesburg, South Africa; 11 CEPREF ART Program, Abidjan, Cote d′Ivoire; 12 Fundación Huésped, Buenos Aires, Argentina; 13 University of Abuja Teaching Hospital, Abuja, Nigeria; 14 USAID AMPATH, Eldoret, Kenya; 15 Indiana University, Indianapolis, Indiana, United States of America; 16 Faculty of Medicine Ramathibodi Hospital, Mahidol University, Bangkok, Thailand; 17 Research Department, Infections Diseases Institute, Kampala, Uganda; 18 University Bordeaux Segalen, ISPED, Bordeaux, France; 19 TREAT Asia, amfAR/The Foundation for AIDS Research, Bangkok, Thailand; 20 Vanderbilt University School of Medicine, Nashville, Tennessee, United States of America; Archivel Farma; Fundació Institut d′Investigació en Ciències de la Salut Germans Trias i Pujol, Universitat Autònoma de Barcelona, CIBERES, Spain

## Abstract

**Objectives:**

In resource-constrained settings, tuberculosis (TB) is a common opportunistic infection and cause of death in HIV-infected persons. TB may be present at the start of antiretroviral therapy (ART), but it is often under-diagnosed. We describe approaches to TB diagnosis and screening of TB in ART programs in low- and middle-income countries.

**Methods and findings:**

We surveyed ART programs treating HIV-infected adults in sub-Saharan Africa, Asia and Latin America in 2012 using online questionnaires to collect program-level and patient-level data. Forty-seven sites from 26 countries participated. Patient-level data were collected on 987 adult TB patients from 40 sites (median age 34.7 years; 54% female). Sputum smear microscopy and chest radiograph were available in 47 (100%) sites, TB culture in 44 (94%), and Xpert MTB/RIF in 23 (49%). Xpert MTB/RIF was rarely available in Central Africa and South America. In sites with access to these diagnostics, microscopy was used in 745 (76%) patients diagnosed with TB, culture in 220 (24%), and chest X-ray in 688 (70%) patients. When free of charge culture was done in 27% of patients, compared to 21% when there was a fee (p = 0.033). Corresponding percentages for Xpert MTB/RIF were 26% and 15% of patients (p = 0.001). Screening practices for active disease before starting ART included symptom screening (46 sites, 98%), chest X-ray (38, 81%), sputum microscopy (37, 79%), culture (16, 34%), and Xpert MTB/RIF (5, 11%).

**Conclusions:**

Mycobacterial culture was infrequently used despite its availability at most sites, while Xpert MTB/RIF was not generally available. Use of available diagnostics was higher when offered free of charge.

## Introduction

Tuberculosis (TB) is the most common opportunistic infection in HIV-infected patients in resource-limited settings and associated with high mortality in patients who start antiretroviral combination therapy (ART) [1], [2]. In high TB-incidence settings, TB may frequently be present at ART initiation, but not always diagnosed, and contributes significantly to mortality in the first weeks and months of ART [3], [4]. One risk factor for early mortality is an unmasking or paradoxical immune reconstitution syndrome that may occur after ART start, particularly in severely immunosuppressed patients [1], [5]–[8].

The overall incidence of TB is substantially reduced with long-term ART in both adults and children [2], [9]–[11] but additional interventions are needed to control TB in HIV-infected patients including screening, improved diagnosis and preventive therapy [12]–[14]. TB screening and diagnosis before starting ART is important, since the initiation of ART shortly after the initiation of TB treatment improves survival, particularly in patients with low CD4 cell counts [15]–[17].

In a survey of 15 ART programs in 12 countries in Africa, the Caribbean, Central and South America, as well as Asia, we previously showed that the capacity of programs to diagnose TB was often limited in 2008 [18]. Since then, new diagnostic tools for rapid TB diagnosis have been introduced and have shown promising results [19], [20]. In 2012, we undertook a large scale survey on the integration of TB and HIV services, diagnostic, screening, preventive and treatment practices within the International epidemiologic Databases to Evaluate AIDS (IeDEA) collaboration. Here, we report the results on the availability and use of TB diagnostics across adult ART programs in low- and middle-income countries, using both program-level and patient-level data.

## Methods

IeDEA includes networks of ART programs in low- and middle-income countries from Africa, Asia, the Caribbean, Central and South America, as well as North America [21]–[23]. Data are collected at each site as part of routine monitoring at program enrollment and each follow-up visit. We surveyed ART sites in low- and middle-income countries participating in IeDEA across Africa, Asia, the Caribbean, Central and South America between March 1 and July 1, 2012. A total of 71 sites treating adults or children were invited and 58 sites (81.7%) participated, including 11 pediatric sites treating children only. See File S1 and Table S1 in Supporting Information for a list of investigators and sites.

### Data collection

Representatives from participating IeDEA regions and the TB and pediatric working groups of IeDEA developed this project. The survey was written in English, translated into French and Spanish, and pilot-tested in both languages. Study data were collected and managed using REDCap (Research Electronic Data Capture) electronic data capture tools (see https://redcap.vanderbilt.edu/) [24]. The survey consisted of three components: *Section A* was an online questionnaire on integration of services, diagnostic, screening, preventive and treatment practices related to TB at the sites; *Section B* included eight clinical scenarios on TB and HIV management to assess the practices of treating clinicians; and *Section C* requested routinely collected data on consecutive TB patients seen during the study period.

*Section A* was completed by local data managers or medical staff. Data collected included site characteristics (level of care, urban or rural setting, number of HIV patients actively followed-up, number of TB cases seen), diagnostic and screening practices, infection control measures, treatment schemes, prevention and prophylaxis practices, integration of HIV and TB care, costs to patients and TB case definitions. Availability was assessed as access to the provider on site, within 5 km, within 10 km, within 20 km, within 30 km, within 40 km, within 50 km, more than 50 km, or not available. In *Section B*, treating physicians and clinical officers at the sites were asked to address clinical scenarios for hypothetical TB patients presenting at their clinic (Table S2). Treating physicians were instructed that the answers should reflect current practices at their treatment program and not necessarily the best practice available. *Sections A* and *B* consisted of one questionnaire per site. In *Section C*, we requested data on HIV-infected TB patients consecutively seen from the start of the study period on March 1 through to July 1, 2012, up to a maximum of 50 patients per site. TB cases were defined based on the local case definition; all received anti-TB treatment. Collected data included age, sex, pregnancy status at time of TB diagnosis, date of enrollment into HIV care, date of TB diagnosis, start date of TB treatment, prescribed TB regimen, start date of ART, CD4 cell count, WHO clinical stage at ART start, date of any previous TB episode, type of screening, type of episode (new case, relapse, treatment after failure, etc.), disease manifestation, sputum microscopy result, culture result, nucleic acid amplification test result, clinical signs and symptoms (coughing, night sweats, fever, weight loss), use of tuberculin skin testing (TST) and isoniazid preventive therapy (IPT). Seven sites from four regions (Asia-Pacific, Caribbean-Central-South America, Central Africa, Southern Africa) did not contribute individual-level patient data because the data collection was not covered by the local IRB approval (four sites), or because of logistical reasons (three sites).

### Statistical analyses

The present analysis included ART programs treating patients aged 16 years or older, and focused on TB diagnostics and screening practices. Descriptive statistics were used to compare characteristics of sites and TB patients. Variables of interest included program-level characteristics (such as level of care, setting, cumulative HIV cohort size, or availability of diagnostics), clinical decision making using hypothetical clinical scenarios (such as decision to conduct sputum smear microscopy, culture, or chest X-ray), and individual-level patient data (such as age, sex, WHO clinical stage, on ART or not when TB treatment started, use of sputum smear microscopy, culture, Xpert MTB/RIF, or chest X-ray to diagnose TB). We distinguished between the availability of diagnostics (“access”) and how diagnostic tools were used at the patient level at sites with access to the tools (“use”). We assumed that there was access to a tool if it was available on or off site. We used χ2 tests or Fisher's exact tests to assess differences between groups in binary variables and the Wilcoxon rank sum test for continuous variables. Results are presented overall and stratified by IeDEA region. All analyses were performed in Stata version 11.2 (Stata Corporation, College Station, TX, USA).

### Data sharing statement

The data held by the IeDEA consortium are available to other investigators, based on a concept note describing the planned analysis which was approved by the regional Steering Groups and, if analyses involve several regions, by the Executive Committee of IeDEA. Further details are provided at www.iedea.org/welcome-to-iedea/working-groups/concept-sheets/iedea-multi-region-concepts-principles-and-procedures.

### Ethics statement

Data were collected through IeDEA cohorts. Ethics committees and/or Institutional Review Boards in all host countries approved the collection and transfer of anonymized data (see File S2 in Supporting Information for a complete list). Where requested per local regulations informed consent was provided. In addition, the Vanderbilt University Health Science Committee, Nashville, Tennessee (USA), the Ethics Committee of the University of Bern (Switzerland), and the University of Cape Town (South Africa) approved the analyses of these observational data for this specific project.

## Results

### Program and patient characteristics

**Figure 1:**
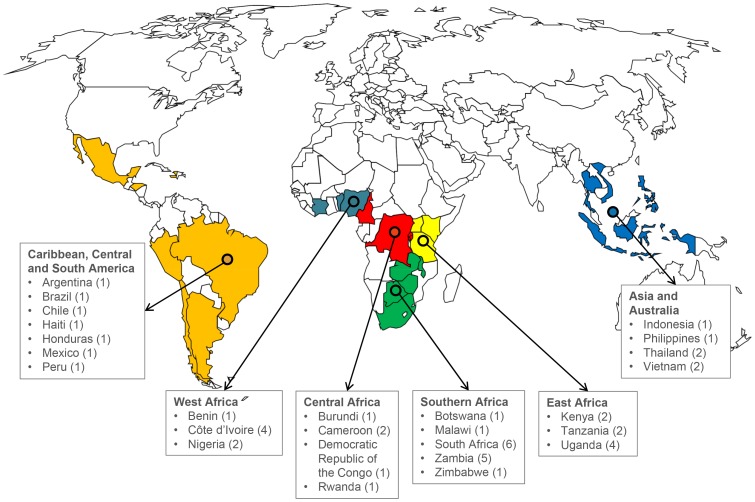
Geographical distribution of the 47 ART programs the International epidemiologic Databases to Evaluate AIDS (IeDEA) collaboration, which treat adults in lower income countries and participated in the survey project. The regions correspond to the IeDEA regions. Countries with at least one site completing *Section A* of the questionnaire are shown. Numbers in parentheses indicate the number of adult ART programs included in the analysis.

Forty-seven ART sites from 26 countries treating adult HIV patients were included (see map in Figure 1 and list in Table S1). These sites treat more than 250,000 HIV-infected adult patients, with almost 18,000 new TB cases diagnosed each year. Twenty-four (51.1%) sites were tertiary care centers and 15 (31.9%) were treating adults only (Table 1). Twenty-eight (59.6%) sites had a specialized TB clinic or ward on site with dedicated staff, nine (19.1%) sites had no specialized clinic, and 10 (21.3%) sites referred TB patients elsewhere. Data on 987 adult TB patients from 40 sites were available. In seven sites, investigators were unable to collect patient data for this survey or no TB cases were seen during the study period. The median age of TB patients was 34.7 years (interquartile range [IQR] 29.4–40.9) and 442 patients (44.8%) were female. The median CD4 cell count at ART initiation was 127 cells/mm^3^ (IQR 42–250), and 128 (13.0%) patients had a history of previous TB. The median time between TB treatment and ART initiation was 37 days (IQR 15–124). The characteristics of patients are presented in Table S3.

**Table 1:** Characteristics of 47 adult ART programs from lower income countries and 987 adult patients seen during the study period in the programs participating in the study.

Characteristic	*All*	Asia Pacific	Caribbean-Central-South America	Central Africa	East Africa	Southern Africa	West Africa
**ART program characteristics**							
Number of sites, n	*47*	6	7	5	8	14	7
Number of countries, n	*26*	4	7	4	3	5	3
Setting, n (%)							
Urban	*38 (80.8)*	6 (100)	7 (100)	5 (100)	4 (50.0)	10 (71.4)	6 (85.7)
Peri-urban	*7 (14.9)*				2 (25.0)	4 (28.6)	1 (14.3)
Rural	*2 (4.3)*				2 (25.0)		
Level of care, n (%)							
Primary	*15 (31.9)*	1 (16.7)			2 (25.0)	10 (71.4)	2 (28.6)
Secondary	*8 (17)*	0	1 (14.3)	2 (40)	1 (12.5)	2 (14.3)	2 (28.6)
Tertiary	*24 (51.1)*	5(83.3)	6 (85.7)	3 (60)	5 (62.5)	2 (14.3)	3 (42.8)
Treating adults and children, n (%)							
Adults and children	*32 (68.1)*	1 (16.7)	3 (42.9)	5 (100)	6 (75.0)	12 (85.7)	5 (71.4)
Adults only	*15 (31.9)*	5 (8.33)	4 (57.1)	0	2 (25.0)	2(14.3)	2 (28.6)
Cost model							
Full payment by the patient	*2 (4.3)*						2 (28.6)
Cost sharing (partial payment)	*19 (40.4)*	3 (50)	2 (28.6)	4 (80)	2 (25)	3 (21.4)	5 (71.4)
Available at no cost for the patient	*23 (48.9)*	2 (33.3)	5 (71.4)	1 (20)	6 (75)	9 (64.3)	0
Other	*3 (6.4)*	1 (33.3)				2 (14.3)	0
Cumulative number of patients enrolled 1, n	*251,377*	8,861	19,029	11,977	103,954	76,767	30,789
New TB cases per year, n	*17,748*	296	1,693	606	2,134	12,378	641
Pre-ART TB (on average) 2, median, %	*22.9*	26	33.9	37.8	10.4	18.9	22.7
**Patient characteristics**							
No. of patients	*987*	35	71	47	255	481	98
Age at start of ART, median (IQR), years	*34.7 (29.4*–*41)*	32.7 (28.6–37.2)	34 (27.3–42.4)	37.1 (30.1–44.6)	35.2 (29.3–42.3)	34.4 (29.1–39.6)	36.2 (31.2–42.2)
Female sex, n (%)	*442 (44.8)*	8 (22.9)	29 (40.8)	23 (40.8)	119 (46.7)	210 (43.7)	53 (54.1)
Site of disease, n (%)							
Pulmonary	*685 (69.4)*	24 (68.6)	56 (78.9)	23 (48.9)	198 (77.6)	328 (68.2)	56 (57.1)
Extrapulmonary	*293 (29.7)*	11 (31.4)	12 (16.9)	20 (42.6)	57 (22.3)	152 (31.6)	41 (41.8)
Unknown	*9 (0.9)*	0	3 (4.2)	4 (8.5)	0	1 (0.2)	1 (1)
Type of TB patient, n (%)							
New case	*863 (87.4)*	25 (71.4)	60 (84.6)	37 (78.7)	236 (92.6)	413 (85.9)	92 (93.9)
Relapse	*78 (7.9)*	5 (14.3)	6 (8.4)	1 (2.1)	13 (5.1)	47 (9.8)	6 (6.1)
Treatment after default	*11 (1.1)*	0	2 (2.8)	0	3 (1.2)	6 (1.2)	0
Other	*23 (2.3)*	0	0	9 (19.1)	1 (0.4)	13 (2.7)	0
CD4 cell count (cells/µl) 1							
At time of ART start, median (IQR)	*127 (42*–*250)*	58 (21–186)	192.5 (71–355)	148 (49–336)	105 (31–271)	113 (42–217)	163.5 (73–281)
* Missing observations, n (%)*	*158 (16.1)*	*2 (5.7)*	*13 (18.3)*	*12 (25.6)*	*43 (16.9)*	*84 (17.5)*	*4 (4.1)*
WHO clinical stage 1, n (%)							
I and II	*58 (6.3)*	0	3 (4.6)	1 (2.3)	19 (7.5)	15 (3.5)	20 (21.0)
III and IV	*863 (93.7)*	35 (100)	63 (94.4)	42 (97.7)	234 (92.5)	414 (96.5)	75 (79.0)
*Missing observations, n*	*66 (6.7)*	0	5 (7)	4 (8.6)	2 (0.8)	52 (10.8)	3 (3.1)
Previous history of TB, n (%)	*180 (18.2)*	0	49 (69)	11 (23.4)	41 (16.1)	56 (11.6)	23 (23.5)
Delay between TB diagnosis and TB treatment, n (%)							
Within 2 days	*810 (82.1)*	23 (65.7)	60 (84.5)	47 (100)	217 (85.1)	401 (83.4)	62 (63.3)
More than 2 days	*177 (17.9)*	12 (34.3)	11 (15.5)		38 (14.9)	80 (16.6)	36 (36.7)
Median delay between TB treatment and ART 2, days, (IQR)	*37 (15*–*124)*	18.5 (11–30)	17 (14–68)	33 (27–127)	63 (31–150)	41.5 (17–129)	36 (32–85)
TB treatment before enrolment to ART	*388 (39.1)*	26 (74.3)	41 (57.7)	15 (31.9)	51 (20.0)	246 (51.1)	9 (9.2)

ART, antiretroviral treatment; TB, tuberculosis; SMS: Short Message Service.

1 followed-up at time of survey.

2 proportion of adults with previous history of TB before starting ART.

### Availability of diagnostics

Sputum smear microscopy and chest X-ray were available at all sites (Table 2). Eighteen (38.3%) sites reported using two sputum samples collected on the spot and early in the morning, 16 (34.0%) used three sputum samples, 12 (25.5%) sites used at least one sputum per patient, and 18 (38.3%) sites used other procedures. Thirty-two sites (68.1%) had conventional chest X-ray and 7 (14.9%) digital X-ray. TST was available in 29 (61.7%) sites. Mycobacterial culture was available in 44 (93.6%) sites (Table 2). The most frequently available culture systems were solid media based culture in 16 sites (36.4%) and liquid culture (MGIT 960, Beckton Dickinson, USA) in 15 sites (34.1%). The semi-automated molecular diagnostic assay Xpert MTB/RIF (Cepheid, USA) was available in about half of the sites (23 sites, 48.9%). The average turnaround time for a TB diagnosis was reported to range between 3 and 4 days (median 3 days, range 1–14) in smear-positive cases and 8–9 days in smear-negative cases (median 7 days, range 1–30). Details on the availability and use of diagnostics are presented in Table S4.

**Table 2:** Program-level characteristics associated with the availability of sputum smear microscopy, culture, chest X-ray, Xpert MTB/RIF, and tuberculin skin testing (TST) in 47 ART programs treating adults in lower income countries.

Program-level characteristic	Sputum microscopy		Culture		Chest X-ray		Xpert MTB/RIF		TST		
	n (%)	*P* value	n (%)	*P* value	n (%)	*P* value	n (%)	*P* value	n (%)	*P* value	
**Total**	**47 (100)**		**44 (93.6)**		**47 (100)**		**23 (48.9)**		**29 (61.7)**		
IeDEA region		–		0.63		–		0.41		0.001	
Asia-Pacific	6		6 (100)		6		4 (66.7)		5 (83.3)		
Caribbean-Central-South America	7		7 (100)		7		2 (28.6)		6 (85.7)		
Central Africa	5		4 (80.0)		5		1 (20.0)		5 (100)		
East Africa	8		7 (87.5)		8		4 (50.0)		2 (25.0)		
Southern Africa	14		13 (92.9)		14		9 (64.3)		4 (28.6)		
West Africa	7		7 (100)		7		3 (42.9)		7 (100)		
Setting		–		0.62		–		0.89		0.017	
Urban	38		36 (95.7)		38		18 (47.4)		27 (71.0)		
Peri-urban	7		6 (85.7))		7		4 (57.1)		1 (14.3)		
Rural	2		2 (100)		2		1 (50.0)		1 (50.0)		
Level of care		–		0.71		–		0.70		0.32	
Primary	15		14 (93.3)		15		7 (46.7)		7 (46.7)		
Secondary	8		7 (87.5)		8		3 (37.5)		5 (62.5)		
Tertiary	24		23 (95.8)		24		13 (54.2)		17 (70.8)		
Treating adults and children		–		0.22		–		0.68		0.077	
Adults and children	15		15 (100)		15		8 (53.3)		12 (80.0)		
Adults only	32		29 (90.6)		32		15 (46.8)		17 (53.1)		
Cumulative number of adults enrolled in HIV care 1		–		0.65		–		0.27		0.12	
Below 1,000	5		5 (100)		5		3 (60.0)		3 (60.0)		
1,001 to 10,000	37		34 (91.9)		37		16 (43.2)		25 (67.6)		
10,001 to 20,000	5		5 (100)		5		4 (80.0)		1 (20.0)		
New TB cases per year, n		–		0.19		–		0.74		0.014	
Below 100	23		20 (86.7)		23		10 (43.5)		19 (82.6)		
100 to 1,000	18		18 (100)		18		10 (55.6)		8 (44.4)		
>1,000	6		6 (100)		6		3 (50.0)		2 (33.3)		

ART, antiretroviral treatment; TB, tuberculosis; TST, tuberculin skin test.

Chi square tests were used to calculate *P* values.

1 followed-up at time of survey.

TST was less frequently available in sites from East and Southern Africa compared to other regions (25% and 28.6%, versus 83.3% to 100%, *P* = 0.001, Table 2). The availability of all other TB diagnostics varied across regions, but differences did not reach statistical significance. TST was also more frequently available in urban settings compared to peri-urban and rural settings (71.0%, 14.3% and 50.0%; *P* = 0.017), as well as more frequent in smaller sites with fewer TB cases (82.6% in sites with fewer than 100 TB cases per year, compared to 44.4% and 33.3% in sites with 100 to 1,000 and more than 1,000 cases; *P* = 0.014).

### Use of diagnostics

Overall, sputum microscopy was done in 745 (75.7%) patients, mycobacterial culture in 220 (23.9%), Xpert MTB/RIF in 120 (20.6%), and chest X-ray in 688 (69.7%, Table 3). Results were similar when restricting the analysis to the 642 patients with pulmonary TB: 574 patients (83.8%) had sputum microscopy, 137 (21.3%) mycobacterial culture, 83 (21.0%) Xpert MTB/RIF, and 460 patients (67.1%) had a chest X-ray. In sites where diagnostic tools were available, the geographic region, degree of urbanization, level of care and payment models were associated with their use (Table 3). For example, culture was more frequently used in sites from the Caribbean, Central and South America than in sites from East Africa (66.2% versus 2.5%, *P*<0.0001). Mycobacterial culture was also more frequently used in urban settings (27.3% in urban settings versus 15.5% in peri-urban and 4.9% in rural settings, *P*<0.0001).

**Table 3:** Availability and use of tuberculosis (TB) diagnostics in adult TB patients from 47 ART programs in lower income countries.

Program-level characteristic	Total	Sputum microscopy			Culture			Chest X-ray			Xpert MTB/RIF			TST		
		Access n	Used n (%)	*P* value	Access n	Used n (%)	*P* value	Access n	Used n (%)	*P* value	Access n	Used n (%)	*P* value	Access n	Used n (%)	*P* value
**Total**	**987**	**987**	**745 (75.5)**		**919**	**220 (23.9)**		**987**	**688 (69.7)**		**584**	**120 (20.6)**		**380**	**39 (10.3)**	
IeDEA region				<0.0001			<0.0001			<0.0001			<0.0001			<0.0001
Asia-Pacific	35 (3.6)	35	31 (88.6)		35	17 (48.6)		35	33 (94.3)		21	5 (23.8)		21	0	
Caribbean-Central-South America	71 (7.2)	71	61 (85.9)		71	47 (66.2)		71	47 (66.2)		54	10 (18.5)		71	27 (38)	
Central Africa	47 (4.8)	47	19 (40.4)		31	3 (9.7)		47	25 (53.2)		20	0		47	1 (2.1)	
East Africa	255 (25.8)	255	212 (83.1)		244	6 (2.5)		255	165 (65.7)		158	3 (1.9)		49	0	
Southern Africa	481 (48.7)	481	353 (72.9)		440	143 (32.5)		481	341 (70.9)		300	101 (33.7)		94	3 (3.2)	
West Africa	98 (9.9)	98	71 (72.4)		98	4 (4.1)		98	77 (70.6)		31	1 (3.2)		98	8 (8.1)	
Setting				0.012			<0.0001			0.56			0.010			0.26
Urban	754 (76.4)	754	554 (73.5)		697	190 (27.3)		754	520 (69.0)		432	86 (19.9)		358	39 (10.9)	
Peri-urban	192 (19.4)	192	154 (80.2)		181	28 (15.5)		192	140 (72.9)		121	33 (27.3)		12	0	
Rural	41 (4.1)	41	37 (90.2)		41	2 (4.9)		41	28 (6.4)		31	1 (3.2)		10	0	
Level of care				<0.0001			0.015			0.46			<0.0001			<0.0001
Primary	418 (42.0)	418	289 (69.1)		377	73 (19.4)		418	299 (71.5)		230	76 (33.0)		162	5 (3.1)	
Secondary	200 (20.0)	200	154 (77.0)		189	56 (29.6)		200	140 (70)		105	20 (19.0)		78	8 (10.3)	
Tertiary	369 (38.0)	369	302 (81.8)		353	91 (25.8)		369	249 (67.5)		249	24 (9.6)		140	26 (18.6)	
Treating adults and children				0.64			<0.0001			0.13			0.69			<0.0001
Adults and children	735 (74.5)	735	552 (75.1)		667	137 (20.6)		735	522 (71.0)		405	85 (21.0)		133	25 (18.8)	
Adults only	252 (25.5)	252	193 (76.6)		252	83 (32.9)		252	166 (6–5.86)		179	35 (19.6)		247	14 (5.7)	
Cumulative number of adults enrolled in HIV care				0.24			0.57			0.069			<0.0001			0.22
Below 1,000	27 (2.9)	27	21 (77.8)		27	5 (18.5)		27	23 (85.2)		14	0		13	0	
1,001 to 10,000	739 (78.3)	739	541 (73.2)		671	172 (25.6)		739	494 (66.9)		410	107 (26.1)		355	39 (11.0)	
10,001 to 20,000	178 (18.9)	178	141 (79.2)		178	41 (23.0)		178	128 (71.9)		117	13 (11.1)		12	0	
New TB cases per year, n				<0.0001			<0.0001			0.016			<0.0001			0.002
Below 100	280 (28.4)	280	184 (65.7)		212	54 (25.5)		280	198 (70.7)		138	12 (8.7)		214	30 (14.0)	
100 to 1,000	471 (47.7)	471	366 (77.7)		471	88 (18.7)		471	310 (65.8)		331	74 (22.4)		101	1 (1.0)	
>1,000	236 (23.9)	236	195 (82.6)		236	78 (33.4)		236	180 (76.3)		115	34 (29.6)		65	8 (12.3)	

The analysis was restricted to patients from sites with access to the diagnostic tools.

Chi square tests were used to calculate *P* values.

### Costs to patients, availability and use

In about half of sites services were free to patients (23 sites, 48.9%). In the other sites (24 sites, 51.1%), the median cost to patients was 2.1 USD (12 sites, IQR 1.5–3.0) for sputum smear microscopy, 25.0 USD (12 sites, IQR 13.7–40.7) for culture, 7.7 USD (20 sites, IQR 5.3–12.0) for chest radiographs, and 50.0 USD (3 sites, IQR 29.6–129.8) for Xpert MTB/RIF. The availability of TB diagnostics was similar in sites providing services for free compared to sites charging patients. But expensive diagnostics (more than 20.0 USD per test) were more frequently used in sites where patients did not have to pay for it. Mycobacterial culture was used in 26.8% of patients from the 20 sites offering free cultures versus 20.8% in the other sites (p = 0.033, Table 4). The same was true for Xpert MTB/RIF (26.2% versus 15.2%, p = 0.001). In contrast, there was no evidence that diagnostics such as sputum microscopy or chest X-ray were more frequently used in sites providing services free of charge (Table 4).

**Table 4:** Availability and use of tuberculosis (TB) diagnostics in HIV-infected individuals, comparing sites with a cost model of free services to the patients and all other sites with full payment, cost sharing or any other cost model.

Diagnostic	Access to diagnostics in sites, n (%)				Use of diagnostics in patients with access to the diagnostics, n (%)			
	*Total*	Sites with free service	All other sites	*P* value	*Total*	Sites with free service	All other sites	*P* value
					*(n = 987)*	(n = 545)	(n = 442)	
Sputum microscopy	*40*	20 (100)	20 (100)	0.99	*987 (100)*	415 (76.1)	330 (74.6)	0.59
Culture	*37*	17 (85.0)	20 (100)	0.072	*919 (93.1)*	128 (26.8)	92 (20.8)	0.033
Xpert MTB/RIF	*20*	8 (40.0)	12 (60.0)	0.21	*584 (59.1)*	74 (26.2)	46 (15.2)	0.001
Chest X-Ray	*40*	20 (100)	20 (100)	0.99	*987 (100)*	368 (67.5)	320 (72.4)	0.10
Tuberculin skin test	*23*	9 (45.0)	14 (70.0)	0.11	*380 (38.5)*	9 (5.7)	30 (13.8)	0.012

Analysis was restricted to sites which contributed patient data and to patients from sites with access to the diagnostics.

Chi square tests were used to calculate *P* values.

### Screening practices before ART initiation

Forty-four (93.6%) sites reported following national guidelines to screen suspected TB cases. Screening practices for active disease included symptom screening (46 sites, 97.9%), chest X-ray (38 sites, 80.9%), sputum smear microscopy (37 sites, 78.7%), culture (16 sites, 34.0%), TST (11 sites, 23.4%) and Xpert MTB/RIF (5 sites, 10.6%, Table S5). To examine the approach of physicians and clinical officers to screen for TB, we confronted them with the hypothetical scenario of an adult newly diagnosed with HIV. At the 46 sites reporting symptom screening as part of their screening practices, 43 (93.5%) physicians reported choosing symptom to screen for TB. In contrast, less than a quarter of clinicians mentioned using sputum smear microscopy and Xpert MTB/RIF despite the fact that these diagnostics were part of the screening practices at their site.

## Discussion

We surveyed 47 ART sites in a global network of HIV adult treatment programs. We found that the use of mycobacterial culture for TB diagnosis was low despite its availability at most sites, while Xpert MTB/RIF was both infrequently available and used. Programs providing services free of charge were associated with higher levels of use of the available diagnostic tools. Our study builds on a smaller survey of the ART in Lower Income Countries (ART-LINC) collaboration in 2008, which showed that the availability of TB diagnostics varied widely across ART programs [18]. The present study covered many additional sites within the larger IeDEA network across Asia, Africa, the Caribbean and Central and South America and offers a global view on ART programs in the low and middle-income countries heavily affected by the generalized HIV and TB epidemics. In this study, we also collected and analyzed information on individual patients and the clinical decision making by treating physicians and clinical officers. This approach allowed us to study the availability, use, and practice of TB diagnostics in parallel.

We found that TB diagnostics were not systematically used even when available. This was illustrated by sputum microscopy and mycobacterial culture, which were not necessarily used for the diagnosis of pulmonary TB even in sites with access to these diagnostics. The discrepancy between availability and use underlines that programmatic issues are essential to the control of TB in the HIV-positive population and need to be considered when new TB diagnostic and screening tools become available in ART programs. Lin and colleagues recently showed that not only new tools are needed to improve TB control, but that the overall quality of patient care and the reduction of patient loss before diagnosis need to be considered as part of an overall implementation concept [25]. The low use of available tools could also be a reflection of some sites performing culture or sputum microscopy only in selected cases, such as treatment failure or relapse, depending on their guidelines. It could also reflect breakdowns in the supply chain for reagents, lack of local expertise on how to use these tools, adherence to national guidelines, or the cost of the test.

The availability of diagnostic tools was similar in programs providing tests free of charge and programs charging patients, but some diagnostics were more frequently used where no costs to patients were incurred. This was particularly true for the automated molecular diagnostic assay Xpert MTB/RIF. It appears that not only the distance to the diagnostic facility matters [26], but also the costs of these diagnostics to the patients. Even if the price of TB diagnostics such as Xpert MTB/RIF has been reduced [27], the costs to the patients may still be prohibitive in relation to their budget. Patients may face difficult choices when balancing the costs of diagnostics with other demands on the family budget. Having free TB diagnostics appears to be important for the successful implementation of TB control in the HIV-infected population.

Our study has several limitations. The results were based on a survey mainly reflecting the situation during the study period as reported by the site representatives. It is therefore difficult to distinguish between intermittent or consistent availability and use. However, we substantiated our findings by collecting data from patient seen during the study period, and by assessing scenarios to capture the clinical decision making of treating physicians and clinical officers.

The ART programs participating in the IeDEA network may not be representative of all ART programs in a country or region. IeDEA sites tend to be located in urban settings, collect data using electronic medical record systems and many of them participate in additional research projects outside the IeDEA collaboration. Of note, a recent survey of HIV care centers in sub-Saharan Africa within the International Center for AIDS Care and Treatment Programs (ICAP)-Columbia University program showed a lower availability of TB diagnostics such as sputum smear microscopy and culture than in our study [28]. This might be explained by the nature of the ICAP program, which consisted of a larger proportion of smaller sites in rural settings, compared to the IeDEA collaboration, which included larger sites in urban settings [21]. Unfortunately, there are only few studies examining availability and use of TB diagnostics in ART programs in low and middle-income countries, which limits the potential for comparisons with other settings. Another limitation is that the analysis of individual patient data was based on those having been diagnosed with TB, rather than those suspected of having TB, although the latter case was addressed in a hypothetical case scenario. Finally, we cannot exclude that the answers provided by the clinicians in the scenarios strictly represented the current practice in their ART program.

In conclusion, we found that access to and use of TB diagnostics is still limited in ART programs and that tests are not systematically used, even when available. Improved diagnostic capacities are necessary for better patient care, to increase TB case retention, reduce delays before treatment initiation, and thereby reducing the risk of onward transmission. Our study suggests that the availability of diagnostics alone does not automatically lead to their use at the patient level, particularly if not free of charge to patients.

Our report underlines the challenges of implementing TB diagnostics in the field. Therefore, when implementing TB diagnostics, our study suggests that it is crucial to consider the affordability of diagnostic tests, the supply chain of reagents, training of laboratory staff, short distances to diagnostic facilities, and prompt access to TB care for diagnosed patients [25], [29]. Further studies need to focus on the barriers preventing access to TB diagnostics and their adequate use. This may have implications for the situation in ART programs as well as TB care centers outside of ART programs. In the meantime, we will continue to monitor availability and use of diagnostics in ART programs in resource limited settings.

## Supporting Information

Table S1 **List of all ART programs participating in the survey and which completed at least** ***Section A*** **(n = 58), including programs treating adults, children, or both.** Sites marked with a star are programs treating children only and were therefore excluded from the present analysis.
(DOC)

Table S2 **Eight hypothetical cases typical for different clinical situations in the context of HIV and tuberculosis management.**
(DOC)

Table S3 **Characteristics of adult tuberculosis (TB) patients seen during the study period from antiretroviral (ART) programs in lower income countries, overall and stratified by IeDEA regions.**
(DOC)

Table S4 **Availability of TB diagnostics in 47 adult ART programs in lower income countries, by IeDEA regions.**
(DOC)

Table S5 **Diagnostic tools as part of the screening practices to diagnose active TB in HIV-infected individuals before starting ART in 47 adult ART programs in lower income countries, overall and stratified by IeDEA regions.**
(DOC)

File S1 **IeDEA tuberculosis working group and participating IeDEA sites and investigators.**
(DOC)

File S2 **Ethics statement including a full list of Ethics Committees and/or Institutional Review Boards.**
(DOC)
