# Supplementary material for: Tuberculosis in Antiretroviral Treatment Programs in Lower Income Countries: Availability and Use of Diagnostics and Screening
Source: PLoS One. 2013 Oct 17;8(10):e77697. doi: 10.1371/journal.pone.0077697 (PMC3798412; doi:10.1371/journal.pone.0077697)
Supplement: Table S1 — List of all ART programs participating in the survey and which completed at least Section A (n = 58), including programs treating adults, children, or both. Sites marked with a star are programs treating children only and were therefore excluded from the present analysis. (DOC) [file pone.0077697.s001.doc]

**Table S1.** List of all ART programs participating in the survey and which completed at least *Section A* (n=58), including programs treating adults, children, or both. Sites marked with a star are programs treating children only and were therefore excluded from the present analysis.

| ***IeDEA region*** | ***Country*** | ***Facility name*** | ***Number of adult HIV patients followed-up ^1^*** | ***Number of new TB cases per year ^2^*** | **Number of TB cases per 100,000 population in the region ^3^** |
| --- | --- | --- | --- | --- | --- |
| Asia/Pacific | Indonesia | Bali Adults | 1943 | 100 | 189 |
|  | *Indonesia* | *Bali Pediatric ** |  |  |  |
|  | *Malaysia* | *Penang Hospital ** |  |  | 65 |
|  | Philippines | RITM | 800 | 40 | 275 |
|  | Thailand | HIV-NAT | 1599 | 30 | 93 |
|  | *Thailand* | *Siriraj Hospital ** |  |  | 405 |
|  | Thailand | Ramathibodi Hospital | 2500 | 40 | 21 |
|  | Vietnam | Bach Mai | 669 | 30 | 116 |
|  | *Vietnam* | *Childrens Hospital 1 ** |  |  | 220 |
|  | *Vietnam* | *Childrens Hospital 2 ** |  |  | 220 |
|  | Vietnam | Hanoi Adult | 1350 | 56 | 200 |
| Caribbean, Central and South America | Argentina | Fundación Huésped | 4000 | 50 | 27 |
|  | Brazil | Instituto de Pesquisa Clínica Evandro Chagas | 2500 | 232 | 91.5 |
|  | Chile | Fundación Arriaran | 2417 | 10 | 16 |
|  | Haiti | Les Centres GHESKIO | 6302 | 1335 | 222 |
|  | Honduras | Instituto Hondureño de Seguridad Social | 710 | 6 | 30 |
|  | Mexico | Instituto Nacional de Ciencias Médicas y Nutrición Salvador Zubirán | 1200 | 10 | 14 |
|  | Peru | Instituto de Medicina Tropical Alexander von Humboldt | 1900 | 50 | 110 |
| Central Africa | Burundi | CHUK Bujumbura | 3545 | 30 | 192 |
|  | Cameroon | Hopital General | 2500 | 128 | 999 |
|  | Cameroon | Hopital Militaire de Yaounde | 3000 | 295 | 999 |
|  | DR Congo | Kalembe Lembe Hospital | 1733 | 80 | 372 |
|  | Rwanda | Military Hospital | 1199 | 73 | 117 |
| East Africa | Kenya | AMPATH | 60202 | 986 | 264 |
|  | Kenya | FACES | 5734 | 114 | 326 |
|  | Tanzania | Morogoro | 2523 | 185 | 153 |
|  | Tanzania | Tumbi | 2383 | 78 | 189 |
|  | Uganda | Makarere | 10076 | 350 | 209 |
|  | Uganda | Masaka | 8115 | 252 | 330 |
|  | Uganda | Mbarara | 8899 | 120 | 209 |
|  | Uganda | Rakai | 6022 | 35 | 209 |
| Southern Africa | Botswana | Independent Surgery | 2400 | 30 | 682 |
|  | Malawi | Lighthouse (Martin Preuss) | 15312 | 3200 | 219 |
|  | South Africa | Desmond Tutu | 5206 | 300 | 877 |
|  | South Africa | Khayelitsha | 6190 | 1501 | 1000 |
|  | South Africa | McCord | 4032 | 231 | 843 |
|  | South Africa | Rahima Moosa | 1200 ^4^ | 30 | 981 |
|  | *South Africa* | *Red Cross ** |  |  | 877 |
|  | South Africa | Themba Lethu | 16500 | 1950 | 452 |
|  | South Africa | Tygerberg | 1179 | 298 | 877 |
|  | Zambia | Chawama | 4518 | 1216 | 1007 |
|  | Zambia | Chelstone | 4359 | 829 | 852 |
|  | Zambia | Chilenje | 3568 | 804 | 903 |
|  | Zambia | George | 4605 | 580 | 417 |
|  | Zambia | Matero | 6019 | 1332 | 1284 |
|  | Zimbabwe | Newlands | 2849 | 77 | 633 |
| West Africa | Bénin | CNHU Cotonou | 2235 | 40 |  |
|  | *Burkina Faso* | *CHU Ouagadougou ** | *2648* |  | 200 |
|  | Côte d’Ivoire | CePReF | 3705 | 80 | 230 |
|  | *Côte d’Ivoire* | *CHU Cocody ** |  |  | 230 |
|  | *Côte d’Ivoire* | *CHU Yopougon ** |  |  | 230 |
|  | Côte d’Ivoire | CIRBA Adultes | 2317 | 30 | 230 |
|  | *Côte d’Ivoire* | *CIRBA Enfants ** |  |  | 230 |
|  | Côte d’Ivoire | CNTS | 1582 | 21 | 230 |
|  | Côte d’Ivoire | MTCT+ Adult and Children | 722 | 11 | 230 |
|  | Nigeria | UATH | 5600 | 308 | 68 |
|  | Nigeria | UBTH | 4586 | 151 | 627 |
|  | *Senegal* | *Albert Royer ** |  |  |  |

ART, antiretroviral treatment; TB, tuberculosis

^1^ Total number of adult HIV-infected patients followed-up at time of survey

^2^ Approximate number of newly detected adult TB cases (all TB forms) per year seen at the ART programs, most recent data

^3^ Local TB incidence in the region of the ART program: number of TB cases (all TB forms) per 100,000 population, most recent data within the last two years

^4^ HIV-infected women attending antenatal or delivery care per year
