# Supplementary material for: Tuberculosis in Antiretroviral Treatment Programs in Lower Income Countries: Availability and Use of Diagnostics and Screening
Source: PLoS One. 2013 Oct 17;8(10):e77697. doi: 10.1371/journal.pone.0077697 (PMC3798412; doi:10.1371/journal.pone.0077697)
Supplement: Table S2 — Eight hypothetical cases typical for different clinical situations in the context of HIV and tuberculosis management. (DOC) [file pone.0077697.s002.doc]

**Table S2.** Eight hypothetical cases typical for different clinical situations in the context of HIV and tuberculosis management.

| **No.** | **Scenario** | **Options (several answers possible)** |
| --- | --- | --- |
| 1 | "Adult patient presents with coughing for more than three weeks, fever, progressive weight loss. Diagnostics: two sputum are found to be microscopically positive for acid-fast bacilli, HIV serology test positive, CD4 cell count < 200." | - Scenario not applicable to our site - Immediate TB treatment - ART initiation 4 weeks after TB treatment start - ART initiation after intensive phase of TB treatment - Different procedure if pregnant women - Other comments |
| 2 | “Adult HIV-infected patient presents with smear-confirmed relapse of pulmonary TB 4 months after completing TB treatment for previous episode and declared cured/treatment completed.” | - Scenario not applicable to our site - Change to retreatment regimen - Prolongation with the same treatment regimen - Culture und drug resistance testing - Nucleic acid amplification test (any method) for drug resistance testing - No actions - Other comments |
| 3 | “Adult HIV-infected patient presents with failure to become smear-negative after 5 months of TB treatment.” | - No actions - Treatment change - Prolongation of treatment (no treatment change) - Culture und drug resistance testing - Nucleic acid amplification test (any method) for drug resistance testing - Transfer to referral center - Other comments |
| 4 | “Adult patient newly diagnosed with HIV arrives in your clinic.” | - Scenario not applicable to our site - No actions - Symptom screening for TB (cough, seats, fever, etc.) - TST - Chest X-Ray - Sputum smear microscopy - Culture - Culture only if smear-negative - Xpert MTB/RIF - Urine lipoarabinomannan assay - Fine needle aspiration - IPT if active TB excluded - IPT if active TB excluded and TST positive - CD4 cell count - Other comments |
| 5 | “An infant (younger than 12 months) with clinical symptoms suspicious for active TB presents at your clinic.” | - Scenario not applicable to our site - No actions - No diagnostic measures, start TB treatment - Refer patient immediately - TST - Gastric lavage, with smear microscopy only - Gastric lavage, with smear microscopy and culture - Induced sputum, with smear microscopy only - Induced sputum, with smear microscopy and culture - Chest X-ray - Fine needle aspiration - “String test” - Xpert MTB/RIF - Other comments |
| 6 | “A child (older than 1 year) with clinical symptoms suspicious for active TB (cough for more than 3 weeks, fever, weight loss not responding to standard anti-bacterial and/or anti-malarial treatment) presents at your clinic.” | - Scenario not applicable to our site - No actions - No diagnostic measures, start TB treatment - TST - Gastric lavage, with smear microscopy only - Gastric lavage, with smear microscopy and culture - Induced sputum, with smear microscopy only - Induced sputum, with smear microscopy and culture - Chest X-ray - Fine needle aspiration - “String test” - Xpert MTB/RIF - Other comments |
| 7 | "Patient (adult or child) newly diagnosed with TB (sputum smear-positive, unknown drug resistance profile) lives in a household of several members." | - Scenario not applicable to our site - No actions - Contact tracing - IPT for all household members if TB excluded - IPT for adults in the household if TB excluded - IPT for HIV-infected individuals in the household if TB excluded - IPT for infants <12 months in the householf if TB excluded - IPT for children <5 years in the household if TB excluded - IPT for pregnant women in household if TB excluded - Assess for active TB - Other comments |
| 8 | "HIV-infected pregnant woman presents at your clinic with clinical symptoms suspicious for TB (cough for more than 3 weeks, fever, weight loss)." | - Scenario not applicable to our site - Shielded chest X-ray - TST - Molecular tests (e.g. Xpert MTB/RIF) - Asking about contact history of TB case - Different TB treatment regimen than for non-pregnant individuals if active TB - Immediate start of TB treatment if active TB - TB treatment deferred until post-partum if active TB - Preferred TB treatment regimen 2HRE 5RH - TB treatment regimen other than 2HRE 5RH is preferred - If MDR TB there is a specific TB treatment regimen - IPT is given in any case if active TB excluded - IPT is given only when positive contact history of TB case and active TB excluded - IPT is deferred until post-partum if active TB excluded - IPT is deferred until postpartum regardless of contact history of TB case - Supplementation of vitamin B6 when IPT administered - In case of active TB contact tracing of own children or children in the same householf for evaluation of IPT - No specific contact tracing of children in case of active TB - Discourage the mother from breast feeding - Recommend breast feeding to the mother - Other comments |

Children are defined as <15 years

ART, antiretroviral therapy; IPT, isoniazid preventive therapy; TB, tuberculosis; TST, tuberculin skin test; 2HRE 5RH, 2 months with isoniazid, rifampicin and ethambutol, followed by 5 months of isoniazid and rifampicin
