# Supplementary material for: Tuberculosis in Antiretroviral Treatment Programs in Lower Income Countries: Availability and Use of Diagnostics and Screening
Source: PLoS One. 2013 Oct 17;8(10):e77697. doi: 10.1371/journal.pone.0077697 (PMC3798412; doi:10.1371/journal.pone.0077697)
Supplement: Table S3 — Characteristics of adult tuberculosis (TB) patients seen during the study period from antiretroviral (ART) programs in lower income countries, overall and stratified by IeDEA regions. (DOC) [file pone.0077697.s003.doc]

**Table S3.** Characteristics of adult tuberculosis (TB) patients seen during the study period from antiretroviral (ART) programs in lower income countries, overall and stratified by IeDEA regions.

| **Characteristic** | *All*  *(n=987)* | Asia Pacific  (n=35) | Caribbean-Central-South America  (n=71) | Central Africa  (n=47) | East Africa  (n=255) | Southern Africa  (n=481) | West Africa  (n=98) |
| --- | --- | --- | --- | --- | --- | --- | --- |
| Age at start of ART, median (IQR), years | *34.7 (29.4-41)* | *32.7 (28.6-37.2)* | 34 (27.3-42.4) | 37.1 (30.1-44.6) | 35.2 (29.3-42.3) | 34.4 (29.1-39.6) | 36.2 (31.2-42.2) |
| Female sex, n (%) | *442 (44.8)* | 8 (22.9) | 29 (40.8) | 23 (40.8) | 119 (46.7) | 210 (43.7) | 53 (54.1) |
| Site of disease, n (%) |  |  |  |  |  |  |  |
| Pulmonary | *685 (69.4)* | 24 (68.6) | 56 (78.9) | 23 (48.9) | 198 (77.6) | 328 (68.2) | 56 (57.1) |
| Extrapulmonary only | *293 (29.7)* | 11 (31.4) | 12 (16.9) | 20 (42.6) | 57 (22.3) | 152 (31.6) | 41 (41.8) |
| Patient category, n (%) |  |  |  |  |  |  |  |
| New case | *863 (87.4)* | 25 (71.4) | 60 (84.6) | 37 (78.7) | 236 (92.6) | 413 (85.9) | 92 (93.9) |
| Relapse | *78 (7.9)* | 5 (14.3) | 6 (8.4) | 1 (2.1) | 13 (5.1) | 47 (9.8) | 6 (6.1) |
| Treatment after failure | *4 (0.4)* | 0 | 3 (4.2) | 0 | 0 | 1 (0.2) | 0 |
| Treatment after default | *11 (1.1)* | 0 | 2 (2.8) | 0 | 3 (1.2) | 6 (1.2) | 0 |
| Transfer in | *8 (0.8)* | 5 (14.3) | 0 | 0 | 2 (0.8) | 1 (0.2) | 0 |
| Other | *23 (2.3)* | 0 | 0 | 9 (19.1) | 1 (0.4) | 13 (2.7) | 0 |
| CD4 cell count (cells / µl) ^1^, median (IQR) | *127 (42-250)* | 58 (21-186) | 192.5 (71-355) | 148 (49-336) | 105 (30.5-271) | 113 (42-217) | 163.5 (73-281) |
| *Missing observations, n (%)* | *158 (16.1)* | *2 (5.7)* | *13 (18.3)* | *12 (25.6)* | *43 (16.9)* | *84 (17.5)* | *4 (4.1)* |
| WHO clinical stage ^1^, n (%) |  |  |  |  |  |  |  |
| I/II | *58 (6.3)* | 0 | 3 (4.6) | 1 (2.3) | 19 (7.5) | 15 (3.5) | 20 (21.0) |
| II/III | *863 (93.7)* | 35 (100) | 63 (94.4) | 42 (97.7) | 234 (92.5) | 414 (96.5) | 75 (79.0) |
| *Missing observations* | *66 (6.7)* | 0 | 5 (7) | 4 (8.6) | 2 (0.8) | 52 (10.8) | 3 (3.1) |
| On TB treatment before starting ART | *389 (39.5)* | 26 (74.3) | 41 (57.7) | 15 (31.9) | 52 (20.4) | 246 (51.1) | 9 (9.2) |
| Previous history of TB, n (%) |  |  |  |  |  |  |  |
| Within the last 2 years | *53 (5.4)* | 4 (11.4) | 6 (8.4) | 7 (14.9) | 8 (3.1) | 22 (4.6) | 6 (6.1) |
| More than 2 years ago | *75 (7.6)* | 3 (8.6) | 6 (8.4) | 9 (19.1) | 9 (3.6) | 44 (9.1) | 4 (4.1) |
| No previous history | *748 (75.9)* | 28 (80.0) | 57 (80.3) | 24 (51.1) | 206 (81.1) | 350 (72.8) | 83 (84.7) |
| Unknown | *111 (11.2)* | 0 | 2 (2.9) | 7 (14.9) | 31 (12.2) | 65 (13.6) | 5 (5.1) |
| Received IPT in the past, n (%) | *6 (0.6)* | 0 | 2 (2.8) | 0 | 0 | 4 (0.8) | 0 |
| Sputum microscopy result ^2^, n (%) |  |  |  |  |  |  |  |
| Positive | *312 (31.6)* | 11 (31.4) | 36 (50.7) | 9 (19.1) | 98 (38.4) | 127 (26.4) | 31 (31.6) |
| Negative | *425 (43.1)* | 19 (54.3) | 24 (33.8) | 9 (19.1) | 112 (43.9) | 222 (46.1) | 39 (39.8) |
| Not done | *242 (24.5)* | 4 (11.4) | 10 (14.1) | 28 (59.6) | 43 (16.9) | 130 (27.0) | 27 (27.6) |
| Pending | *8 (0.8)* | 1 (2.9) | 1 (1.4) | 1 (2.1) | 2 (0.8) | 2 (0.4) | 1 (1.0) |
| Culture ^2^, n (%) |  |  |  |  |  |  |  |
| Positive | *133 (13.5)* | 8 (22.9) | 32 (45.1) | 1 (2.1) | 0 | 91 (18.9) | 1 (1) |
| Negative | *67 (6.8)* | 9 (25.7) | 4 (5.6) | 2 (4.3) | 6 (2.3) | 45 (9.4) | 1 (1) |
| Not done | *766 (77.6)* | 18 (51.4) | 24 (33.8) | 44 (93.6) | 249 (97.6) | 337 (70.1) | 94 (95.9) |
| Pending | *21 (2.1)* | 0 | 11 (15.5) | 0 | 0 | 8 (1.7) | 2 (2.0) |
| Xpert MTB/RIF ^2^, n (%) |  |  |  |  |  |  |  |
| Positive | *79 (8.0)* | 5 (14.3) | 7 (9.9) | 0 | 1 (0.4) | 66 (13.7) | 0 |
| Negative | *43 (4.4)* | 0 | 3 (4.2) | 0 | 3 (1.2) | 36 (7.5) | 1 (1) |
| Not done | *863 (87.4)* | 30 (85.7) | 60 (84.5) | 47 (100) | 251 (98.4) | 378 (78.6) | 97 (98.9) |
| Pending | *2 (0.2)* | 0 | 1 (1.4) | 0 | 0 | 1 (0.2) | 0 |
| Chest X-ray ^2^, n (%) |  |  |  |  |  |  |  |
| Positive (suspicious of TB) | *568 (57.6)* | 16 (45.7) | 41 (57.8) | 20 (42.6) | 140 (54.9) | 291 ( 60.5) | 60 (61.2) |
| Negative | *99 (10.0)* | 17 (48.6) | 4 (5.6) | 2 (4.3) | 25 (9.8) | 36 (7.5) | 15 (15.3) |
| Not done | *299 (30.3)* | 2 (5.7) | 24 (33.8) | 22 (46.8) | 90 (35.3) | 140 (29.1) | 21 (21.4) |
| Done, no result | *21 (2.1)* | 0 | 2 (2.8) | 3 (6.4) | 0 | 14 (2.9) | 2 (2.0) |
| Coughing >3 weeks, n (%) | *586 (59.4)* | 25 (71.4) | 39 (54.9) | 34 (72.3) | 208 (81.6) | 203 (42.2) | 77 (78.6) |
| Unknown | *252 (25.5)* | 3 (8.6) | 24 (33.8) | 8 (17) | 19 (7.4) | 195 (40.5) | 3 (3.1) |
| Night sweats, n (%) | *478 (48.4)* | 28 (80) | 20 (28.2) | 27 (57.4) | 172 (67.4) | 179 (37.2) | 52 (53.1) |
| Unknown | *319 (32.3)* | 7 (20) | 34 (47.9) | 10 (21.3) | 35 (13.7) | 218 (45.3) | 15 (15.3) |
| Fever, n (%) | *502 (50.9)* | 32 (91.4) | 35 (49.3) | 31 (66) | 174 (68.2) | 148 (30.8) | 82 (83.7) |
| Unknown | *310 (31.4)* | 2 (5.7) | 23 (32.4) | 7 (14.9) | 41 (16.1) | 233 (48.4) | 4 (4.1) |
| Weight loss, n (%) | *458 (46.4)* | 25 (71.4) | 23 (32.4) | 29 (61.7) | 126 (49.4) | 185 (38.5) | 70 (71.4) |
| Unknown | *331 (33.6)* | 5 (14.3) | 30 (42.2) | 7 (14.9) | 52 (20.4) | 255 (46.8) | 12 (12.2) |
| Delay between TB diagnosis and TB treatment, n (%) |  |  |  |  |  |  |  |
| Within 2 days | *810 (82.1)* | 23 (65.7) | 60 (84.5) | 47 (100) | 217 (85.1) | 401 (83.4) | 62 (63.3) |
| More than 2 days | *177 (17.9)* | 12 ( 34.3) | 11 (15.5) |  | 38 (14.9) | 80 (16.6) | 36 (36.7) |
| Median delay between TB treatment and ART, days, (IQR) | *37 (15-124)* | 18.5 (11-30) | 17 (14-68) | 33 (27-127) | 63 (31-150) | 41.5 (17-129) | 36 (32-85) |
| *Missing observations* | *0* |  |  |  |  |  |  |

^1^ at the start of ART

^2^ at the time of TB diagnosis

^3^ among patients who were on TB treatment before starting ART (n=388)

Abbreviations: ART, antiretroviral therapy; IeDEA, International epidemiological Databases to Evaluate AIDS; IQR, interquartile range; TB, tuberculosis
