# Supplementary material for: Tuberculosis in Antiretroviral Treatment Programs in Lower Income Countries: Availability and Use of Diagnostics and Screening
Source: PLoS One. 2013 Oct 17;8(10):e77697. doi: 10.1371/journal.pone.0077697 (PMC3798412; doi:10.1371/journal.pone.0077697)
Supplement: Table S4 — Availability of TB diagnostics in 47 adult ART programs in lower income countries, by IeDEA regions. (DOC) [file pone.0077697.s004.doc]

**Table S4.** Availability of TB diagnostics in 47 adult ART programs in lower income countries, by IeDEA regions.

| **Test** | *All* | Asia Pacific | Caribbean-Central-South America | Central Africa | East Africa | Southern Africa | West Africa |
| --- | --- | --- | --- | --- | --- | --- | --- |
| n (%) | *(n=47)* | (n=6) | (n=7) | (n=5) | (n=8) | (n=14) | (n=7) |
| **Sputum smear microscopy** |  |  |  |  |  |  |  |
| Availability |  |  |  |  |  |  |  |
| On site | *36 (76.6)* | 5 (83.3) | 7 (100) | 3 (60.0) | 8 (100) | 11 (78.6) | 2 (28.6) |
| Within 5 km | *8 (17)* | 1 (16.7) |  | 2 (40) |  | 1 (7.1) | 4 (57.1) |
| Within 5-20 km | *2 (4.3)* |  |  |  |  | 2 (14.3) |  |
| More than 20 km | *1 (2.1)* |  |  |  |  |  | 1 (14.3) |
| Not available |  |  |  |  |  |  |  |
| Mode of use |  |  |  |  |  |  |  |
| At least one per patient | *12 (25.5)* | 2 (33.3) | 2 (28.6) | 0 | 2 (25) | 5 (35.7) | 1 (14.3) |
| Spot-morning | *18(38.3)* | 2 (33.3) | 4 (57.1) | 0 | 6 (75) | 5 (35.7) | 1 (14.3) |
| Others | *17 (36.2)* | 2 (33.3) | 1 (14.3) | 5 (100) |  | 4 (28.6) | 5 (71.4) |
| Type of staining |  |  |  |  |  |  |  |
| Auramin fluorochrome | *7 (14.9)* |  |  |  | 1 (12.5) | 6 (42.9) |  |
| Ziehl-Neelsen | *31 (66)* | 6 (100) | 5 (71.4) | 4 (80.0) | 6 (75) | 7 (50.0) | 3 (42.9) |
| Both | *9 (19.1)* |  | 2 (28.6) | 1 (20.0) | 1 (12.5) | 1 (7.1) | 4 (57.1) |
| **Mycobacterial culture** |  |  |  |  |  |  |  |
| Availability |  |  |  |  |  |  |  |
| On site | *12 (25.5)* | 4 (66.7) | 6 (85.7) |  | 1 (12.5) | 1 (7.1) |  |
| Within 5 km | *11 (23.4)* | 2 (33.3) | 1 (14.3) | 2 (40.0) | 1 (12.5) | 3 (21.4) | 2 (28.6) |
| Within 5-20 km | *14 (29.8)* |  |  | 2 (40.0) | 1 (12.5) | 8 (57.1) | 3 (42.9) |
| More than 20 km | *7 (14.9)* |  |  |  | 4 (50) | 1 (7.1) | 2 (28.6) |
| Not available | *3 (6.4)* |  |  | 1 (20) | 1 (12.5) | 1 (7.1) |  |
| Type of culture system |  |  |  |  |  |  |  |
| MGIT 960 liquid culture | *15 (34.1)* | 4 (66.7) | 1 (14.3) |  | 5 (71.4) | 5 (33.3) |  |
| BACTEC 460 liquid culture | *4 (9.1)* |  | 1 (14.3) |  | 1 (14.3) | 1 (7.7) | 1 (14.3) |
| Solid media | *16 (36.4)* | 2 (33.3) | 3 (18.9) | 2 (50.0) | 1 (14.3) | 4 (30.8) | 4 (57.1) |
| Other | *9 (20.4)* |  | 2 (28.6) | 2 (50.0) |  | 3 (23.1) | 2 (28.6) |
| **Xpert MTB/RIF** |  |  |  |  |  |  |  |
| Availability |  |  |  |  |  |  |  |
| On site | *13 (27.7)* | 2 (33.3) | 2 (28.6) |  | 3 (37.5) | 5 (35.7) | 1 (14.3) |
| Within 5 km | *2 (4.3)* | 1 (16.6) |  | 1 (20.0) |  |  |  |
| Within 5-20 km | *7 (14.9)* | 1 (14.3) |  |  | 1 (12.5) | 3 (21.4) | 2 (28.6) |
| More than 20 km | *1 (2.1)* |  |  |  |  | 1 (7.1) |  |
| Not available | *24 (51.1)* | 2 (33.3) | 5 (71.4) | 4 (80.0) | 4 (50.0) | 5 (35.7) | 4 (57.1) |
| Mode of use |  |  |  |  |  |  |  |
| All patients | *5 (27.8)* | 1 (33.3) |  |  | 1 (25.0) | 3 (60.0) |  |
| Only when drug resistance suspected | *1 (5.6)* |  |  |  | 1 (25.0) |  |  |
| Patients on treatment failure |  |  |  |  |  |  |  |
| Suspected drug resistance and treatment failure |  | 1 (33.3) | 1 (50.0) |  |  |  | 1 (33.3) |
| Number of specimens used |  |  |  |  |  |  |  |
| Only one clinical specimen | *7 (41.2)* |  | 1 (50.0) | 1 (100) | 2 (66.7) | 3 (60.0) |  |
| Always two clinical specimen | *5 (29.4)* | 2 (66.7) |  |  | 1 (33.3) | 1 (20.0) | 1 (33.3) |
| Second specimen when first negative | *3 (17.6)* | 1 (33.3) | 1 (50.0) |  |  | 1(20.0) |  |
| Other | *2 (11.8)* |  |  |  |  |  | 2 (66.7) |
| **Chest X-ray** |  |  |  |  |  |  |  |
| Availability |  |  |  |  |  |  |  |
| On site | *37 (78.7)* | 5 (83.3) | 7 (100) | 5 (100) | 6 (75.0) | 10 (71.4) | 4 (57.1) |
| Within 5 km | *8 (17.0)* | 1 (16.7) |  |  | 2 (25.0) | 2 (14.3) | 3 (42.9 |
| Within 5-20 km | *2 (4.3)* |  |  |  |  | 2 (14.3) |  |
| More than 20 km | *0* |  |  |  |  |  |  |
| Not available | *0* |  |  |  |  |  |  |
| Type of chest X-ray |  |  |  |  |  |  |  |
| Conventional X-ray | *32 (68.1)* | 2 (33.3) | 4 (57.1) | 3 (60.0) | 5 (62.5) | 12 (85.6) | 6 (85.7) |
| Digital X-ray | *7 (14.9)* | 3 (50.0) | 1 (14.3) |  | 1 (12.5) | 2 (14.3) |  |
| Combination of above | *8 (17.0)* | 1 (16.7) | 2 (28.6) | 2 (40.0) | 2 (25.0) |  | 1 (14.3) |
| **Tuberculin skin test** |  |  |  |  |  |  |  |
| Availability |  |  |  |  |  |  |  |
| On site | *21 (44.7)* | 5 (83.3) | 6 (85.7) | 2 (40.0) | 1 (12.5) | 4 (28.6) | 3 (42.9) |
| Within 5 km | *7 (14.9)* |  |  | 2 (40.0) | 1 (12.5) |  | 4 (57.1) |
| Within 5-20 km |  |  |  |  |  |  |  |
| More than 20 km | *1 (2.1)* |  |  | 1 (20.0) |  |  |  |
| Not available | *18 (38.3)* | 1 (16.7) | 1 (14.3) |  | 6 (75.0) | 10 (71.4) |  |

ART, antiretroviral therapy; IeDEA, International epidemiological Databases to Evaluate AIDS; TB, tuberculosis;
