# Supplementary material for: Tuberculosis in Antiretroviral Treatment Programs in Lower Income Countries: Availability and Use of Diagnostics and Screening
Source: PLoS One. 2013 Oct 17;8(10):e77697. doi: 10.1371/journal.pone.0077697 (PMC3798412; doi:10.1371/journal.pone.0077697)
Supplement: Table S5 — Diagnostic tools as part of the screening practices to diagnose active TB in HIV-infected individuals before starting ART in 47 adult ART programs in lower income countries, overall and stratified by IeDEA regions. (DOC) [file pone.0077697.s005.doc]

**Table S5.** Diagnostic tools as part of the screening practices to diagnose active TB in HIV-infected individuals before starting ART in 47 adult ART programs in lower income countries, overall and stratified by IeDEA regions.

| **Test** | *All* | Asia Pacific | Caribbean-Central-South America | Central Africa | East Africa | Southern Africa | West Africa |
| --- | --- | --- | --- | --- | --- | --- | --- |
| n (%) | *(n=47)* | (n=6) | (n=7) | (n=5) | (n=8) | (n=14) | (n=7) |
|  |  |  |  |  |  |  |  |
| National guidelines for TB screening | *44 (93.6)* | 5 (83.3) | 5 (71.4) | 5 (100) | 8 (100) | 14 (100) | 7 (100) |
| Sputum microscopy | *37 (78.7)* | 5 (83.3) | 7 (100) | 4 (80) | 6 (75) | 10 (71.4) | 5 (71.4) |
| Culture | *16 (34)* | 3 (50) | 6 (85.7) | 1 (20) | 1 (12.5) | 4 (28.6) | 1 (14.3) |
| Xpert MTB/RIF | *5 (10.6)* | 1 (16.7) | 0 | 0 | 1 (12.5) | 2 (14.3) | 1 (14.3) |
| Chest X-ray | *38 (80.8)* | 6 (100) | 7 (100) | 4 (80) | 6 (75) | 10 (71.4) | 5 (71.4) |
| Urine lipoarabinomannan test | *0* | 0 | 0 | 0 | 0 | 0 | 0 |
| Tuberculin skin testing | *11 (23.4)* | 1 (16.7) | 6 (85.7) | 2 (40) | 0 | 1 (7.1) | 1 (14.3) |
| Induced sputum | *10 (21.3)* | 3 (50) | 4 (57.1) | 0 | 1 (12.5) | 2 (14.3) | 0 |
| Gastric lavage | *4 (8.5)* | 0 | 3 (42.9) | 1 (20) | 0 | 0 | 0 |
| Biopsy | *21 (44.7)* | 4 (66.7) | 6 (85.7) | 2 (40) | 4 (50) | 2 (14.3) | 3 (42.9) |
| Contact history with a TB case in family | *39 (83)* | 5 (83.3) | 7 (100) | 4 (80) | 5 (62.5) | 12 (85.7) | 6 (85.7) |
| Symptom screening |  |  |  |  |  |  |  |
| Any symptoms | *46 (97.9)* | 6 (100) | 7 (100) | 5 (100) | 8 (100) | 14 (100) | 6 (85.7) |
| Coughing (any duration) | *42 (89.4)* | 6 (100) | 7 (100) | 5 (100) | 6 (75) | 14 (100) | 4 (57.1) |
| Fever (any duration) | *44 (93.7)* | 6 (100) | 7 (100) | 5 (100) | 7 (87.5) | 14 (100) | 5 (71.4) |
| Night sweats | *45 (95.7)* | 5 (83.3) | 7 (100) | 5 (100) | 8 (100) | 14 (100) | 6 (85.7) |
| Weight loss | *45 (95.7)* | 5 (83.3) | 7 (100) | 5 (100) | 8 (100) | 14 (100) | 6 (85.7) |
| Others | *5 (10.6)* | 1 (16.7) | 1 (14.3) | 0 | 2 (25) | 0 | 1 (14.3) |
|  |  |  |  |  |  |  |  |

ART, antiretroviral treatment; IeDEA, International epidemiological Databases to Evaluate AIDS; TB, tuberculosis
